# Computational Fluid Dynamics Modeling of a Broiler House Microclimate in Summer and Winter

**DOI:** 10.3390/ani12070867

**Published:** 2022-03-29

**Authors:** Erdem Küçüktopcu, Bilal Cemek, Halis Simsek, Ji-Qin Ni

**Affiliations:** 1 Department of Agricultural Structures and Irrigation, Ondokuz Mayıs University, Samsun 55139, Turkey; bcemek@omu.edu.tr; 2 Department of Agricultural and Biological Engineering, Purdue University, West Lafayette, IN 47907, USA; simsek@purdue.edu (H.S.); jiqin@purdue.edu (J.-Q.N.)

**Keywords:** model, CFD, numeric analysis, indoor environment, poultry building, simulation

## Abstract

**Simple Summary:**

Microclimate conditions in broiler housing are significant for maximizing poultry production and ensuring the welfare of the birds. In the present study, we modeled summer and winter microclimates in a mechanically ventilated broiler house. Validation of the simulated values was accomplished through comparison to field measurements. In visual simulations, the results were used to reconstruct microclimate conditions such as stagnant and stress zones of broiler houses. In conclusion, simulation techniques can be used as an alternative method for analyzing poultry house indoor environments.

**Abstract:**

Appropriate microclimate conditions in broiler housing are critical for optimizing poultry production and ensuring the health and welfare of the birds. In this study, spatial variabilities of the microclimate in summer and winter seasons in a mechanically ventilated broiler house were modeled using the computational fluid dynamics (CFD) technique. Field measurements of temperature, relative humidity, and airspeeds were conducted in the house to compare the simulated results. The study identified two problems of high temperature in summer, which could result in bird heat stress and stagnant zones in winter, and simulated possible alternative solutions. In summer, if an evaporative cooling pad system was used, a decrease in temperature of approximately 3 °C could be achieved when the mean air temperature rose above 25 °C in the house. In winter, adding four 500-mm circulation fans of 20-m spacing inside the house could eliminate the accumulation of hot and humid air in the stagnant zones in the house. This study demonstrated that CFD is a valuable tool for adequate heating, ventilation, and air conditioning system design in poultry buildings.

## 1. Introduction

The world population is expected to exceed 8 billion by 2025, meaning that it is crucial to address the issue of meeting food demand [1]. Providing sufficient nutritional intake for the increasing population will be possible by increasing food production qualities and capacities. Aiming to obtain more and higher quality products from the per-unit area in the field has necessitated the application of new production techniques by adopting automation and mechanization systems [2,3,4]. Applying these new technologies in animal production has led broiler farming to become an increasingly important economic sector in Turkey, which represents 2.16% of global chicken meat production, and consequently ranks 10th in the world for chicken meat production, with 2,138,000 tons/year capacity. Poultry meat export also comprises 3.70% of the country’s total exports, at 408,000 tons [5]. Poultry meat production in Turkey is projected to reach 3,350,000 tons by 2025. To achieve this goal, it is necessary to increase modernization in production, expand genetic breeding research, and provide the most suitable environmental conditions for birds. Efforts to increase poultry production and productivity emphasize breeding and feeding research [6,7,8,9,10]. However, achieving the desired level of productivity is impossible if the poultry building’s environmental conditions are inadequate [11].

Poultry building structures differ considerably from other animal facilities because chickens are highly sensitive to temperature and air exchange fluctuations. The primary purpose of a poultry building is to protect the animals from adverse environmental conditions, which would cause mortality or reduced growth and feed efficiency [12,13]. Therefore, poultry buildings should be planned to provide suitable environmental conditions.

In planning and designing poultry buildings, both the optimal environmental conditions (indoor) and the climate data from the location (ambient) are critical factors to consider successful production [14,15]. Productivity is highly dependent on indoor environmental conditions, since birds are very sensitive animals. Failure to provide the ideal brooding conditions reduces profitability, as birds’ growth will be slow, decreasing feed intake, and increasing disease and mortality [16]. In 1920, the average mortality of broilers was approximately 20%. By the end of the 20th century, mortality reduced to 4% with the improvements in nutrition and housing qualities and advanced disease control methods [17]. All these experiences show how critical it is to provide an optimal indoor environment in the poultry building.

One of the most effective methods of determining the environmental conditions of a poultry building is to evaluate the conditions using modeling techniques. Models can be designed to simulate real events with simple approaches. Accurate predictions can be made to obtain desired information about the events. In the simulations, heat and mass balance equations are solved to determine the environmental conditions of the building using many factors such as animal species and age, characteristics of the building, and indoor and ambient conditions [18]. These equations, which cannot be calculated directly using analytical methods, can be solved with numerical methods called computational fluid dynamics (CFD).

The CFD technique can be applied in existing poultry housing or before constructing the building. Determination of the building during the planning and designing stages can save time and cost and provide a visual way to analyze the results. Therefore, CFD has gained popularity in recent years as a result of technological advances. The method has been successfully applied in a wide variety of industrial fields, such as aerospace [19,20], automotive [21,22], and agriculture [23,24,25,26,27,28,29,30,31,32]. These studies have contributed significantly to our understanding; however, no previous studies have sufficiently assessed the indoor environment of mechanically ventilated broiler buildings in different breeding seasons (summer and winter).

In this study, spatial variations between summer and winter for the microclimate in a mechanically ventilated broiler house are simulated for the first time using CFD techniques to understand the reasons for temperature and airspeed distribution shortcomings. Alternative solutions are proposed to enhance the performance of broiler houses.

## 2. Materials and Methods

### 2.1. Broiler House

**Figure 1:**
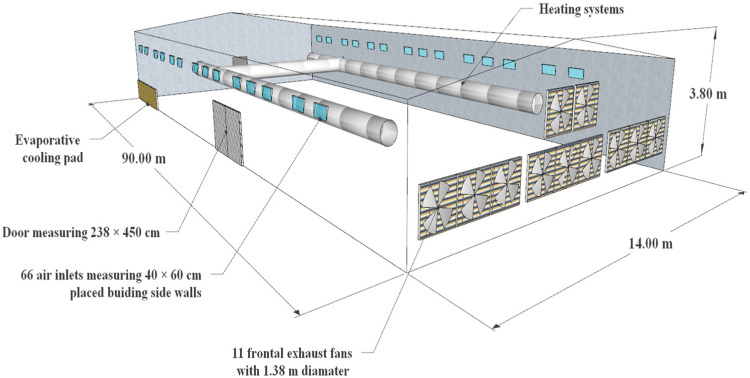
The dimensions of the broiler house.

A field experiment was conducted at a mechanically ventilated broiler house located in Samsun, Turkey (41°70′ N, 36°30′ E). The room dimensions and other properties were as follows. The room was 90 m long and 14 m wide, and the heights of the sidewalls and ridge were 2.70 m and 3.80 m, respectively (Figure 1). The house was oriented in an east–west direction. The east wall had 11 exhaust fans of 1.38-m diameter (EOS53, Termotecnica Pericoli, Albenga, Italy). The sidewalls of the house were equipped with 66 air inlets of 40 cm × 60 cm. A static pressure controller was used to maintain the desired airflow rate in the room. Sandwich panels with an expanded polystyrene insulation material of 5-cm thickness were used to clad the walls and roof of the house. The floor was made of concrete and was usually covered with a 50-mm layer of rice hulls as initial bedding material.

One-day-old broiler chickens of the “Ross 308” commercial hybrid breed were reared to 42 days of age. The number of birds housed were 18,240 and 18,000 during the summer and winter, respectively.

### 2.2. CFD Model and Boundary Conditions

The commercial CFD code Ansys^®^ Fluent (version 13, Fluent Inc., Lebanon, NH, USA) was used to model the spatial variability during the summer and winter seasons in the broiler housing. The 3D geometric model of the house was developed using AutoCAD software (version 2016, Autodesk, San Rafael, CA, USA) on the 40th breeding day of a 42-day breeding period. Hence, summer and winter simulations were applied on 24 August 2019 at 13:47 and 14 March 2018 at 16:45, respectively.

Circular exhaust fans and rectangular air inlets were used with a surface area of 1.49 m^2^ and 0.24 m^2^, respectively. Since the dimensions of the watering and feeding equipment were much smaller than the dimensions of the house, the effects of such equipment on the microclimate were neglected. The birds were considered to be a porous layer that released heat. Porosity was calculated to 90% by considering the number of birds, the house area, the wet volume of each bird, the feather width, and the height of each bird [33]. For the pressure drop calculation, the viscous resistance and inertial resistance coefficients were equal to 1/α = 186 m^−2^ and C2 = 4.4 m^−1^ [34].

Sensible heat production (*SHP*) from the broilers was calculated as a sum of convective and radiant heat loss. The convective heat loss (*C*) was estimated by using the Equation (1) [35]:

(1) $$C=\frac{k\Delta TNu}{d}$$

where *C* is the convective heat loss (W m^−2^); *k* is the thermal conductivity of air (W m^−1^ K^−1^); Δ*T* is the temperature difference between the air and the chicken’s surface (°C); *Nu* is the Nusselt number; and *d* is the characteristic dimension of a chicken (m). The characteristic dimension of a chicken can be calculated from its mass, *W* (kg) as indicated in Equation (2) [36]:

(2) $$d=0.131W^{0.33}$$

The Nusselt number can be calculated as [33]:

(3) $$Nu=2+0.79Re^{0.48}$$

where *Re* is the Reynolds number, which can be calculated as:

(4) $$Re=\frac{\nu d}{\mu }$$

where *ν* is air velocity (m s^−1^), and *μ* is air dynamic viscosity (kg m^−1^ s^−1^). According to McArthur [37], the radiant heat loss, *L_n_* (W m^−2^) can be determined by Equation (5):

(5) $$L_{n}=\frac{\rho c_{p}}{r_{R}}\left(T_{c}-T_{e}\right)$$

where *ρc_p_* is the volumetric specific heat of the air (J m^−3^ K^−1^); *T_c_* is the surface temperature of the feathers (°C); *T_e_* is the surrounding air temperature (°C); and *r_R_* is the radiative resistance (s m^−1^), which can be calculated as:

(6) $$r_{R}=\frac{rc_{p}}{4\sigma {\left(T_{ce}\right)}^{3}}$$

*σ* is the Stefan–Boltzmann constant (W m^−2^ K^−4^), and *T_ce_* is the average temperature of *T_c_* and *T_e_* (K). The total *SHP* from birds was calculated as [38]:

(7) $$SHP=A_{C}\left(C+L_{n}\right)$$

where *SHP* is the total sensible heat production (W bird^−1^), and *A_c_* is the surface area of bird’s coat (m^2^), which can be computed as [38]:

(8) $$A_{C}=0.081W^{0.667}$$

The latent heat production (*LHP*) and moisture production (*MP*) for birds were calculated using the following equations, respectively [39]:

(9) $$LHP=5.73W^{2}-12.34W+8.88$$

(10) $$MP=\frac{LHP}{2450000}$$

where *LHP* is the total latent heat production (W bird^−1^), and *MP* is the moisture production (kg s^−1^). Cellulose pads (Munters, Kista, Sweden) with angles of 60–30° and thickness of 100 mm were used as evaporative pads. The porosity, viscous, and inertial resistances were taken as 94.7%, 3.3 × 10^6^ m^−2^, and 1.13 × 10^−4^ m^−1^, respectively [40].

Simulations were carried out under steady-state conditions. The Boussinessq approximation was employed to consider the buoyancy effect. The SIMPLE algorithm with second-order precision was selected. The renormalization group (RNG) k-ε turbulence model was used to predict the indoor environment of broiler housing because it has been suggested to be more accurate than standard k-ε and realizable k-ε turbulence models [27]. The convergence criterion was fixed to 10^−4^ for the continuity, momentum, and turbulence equations and 10^−6^ for the energy equation. The initial boundary condition for solving the numerical solution is listed in Table 1. The external climatic conditions were monitored and recorded in detail throughout the breeding periods.

### 2.3. Meshing Design

A mesh sensitivity test was achieved to select an optimum mesh number. Four different grid sizes, including grid 1 (0.85 million cells), grid 2 (3.60 million cells), grid 3 (7.50 million cells), and grid 4 (13.80 million cells), were compared with airspeed profiles along the centerline. Relative errors of grids 1, 2, 3, and 4 were 17.50, 6.23, 5.74, and 1.75, respectively. The numerical results were stabilized in this study, and minor differences were found from grid 2 to 3. Moreover, grid 2 required significantly less computing time and power than grid 3. Therefore, the decision was made to use the grid 2 model to solve the problem. The grid 1 and 4 models were not preferred due to their high relative error and long processing time.

### 2.4. Field Measurement

**Figure 2:**
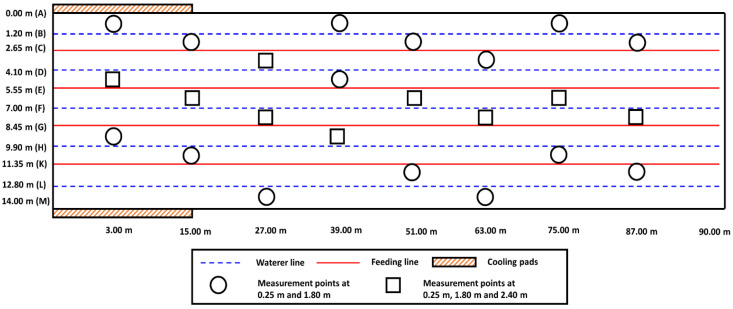
Measurement locations for airspeed, temperature, and relative humidity measurements inside the house.

Measurements were performed at three heights: (i) the height of the flock occupation (0.25 m); (ii) the height of an average adult human (1.80 m); and (iii) the height where highest airspeeds were expected (2.40 m). Fifty-seven measurements were taken in various locations inside the house at the three heights, including twenty-four at 0.25 m, twenty-four at 1.80 m, and nine at 2.40 m (Figure 2).

To monitor the indoor air temperature and relative humidity distribution, fifty-seven data loggers (HT71N, PCE Instruments, Jupiter, FL, USA) with an accuracy of ±0.5 °C for the temperature and ±2.0% for the relative humidity were used. Three hotwire anemometers (PCE-423, PCE Instruments, Jupiter, FL, USA) with an accuracy of ±5% were used to characterize airspeed distributions. The measurements were carried out simultaneously in three parts of the house, starting from the front to the back and then from the back of the house to the front. All the instruments were calibrated before use.

### 2.5. Model Validation

The CFD simulation results were validated by comparing the measured airspeeds, air temperatures, and humidity in the summer and winter seasons. Some evaluation indicators, including fractional bias (*FB*), geometric mean bias (*MG*), geometric mean-variance (*VG*), fraction within a factor of two (*FAC2*), and normalized mean square error (*NMSE*) were used to evaluate the accuracy of the CFD models [41,42,43]. The evaluation indicators were calculated using Equations (11)–(15).

(11) $$FB=2\frac{{\bar{X}}_{mea}-{\bar{X}}_{pre}}{{\bar{X}}_{mea}+{\bar{X}}_{pre}}$$

(12) $$MG=exp\left[\bar{ln\left(\frac{X_{mea}}{X_{pre}}\right)}\right]$$

(13) $$VG=exp\left[\bar{ln{\left(\frac{X_{mea}}{X_{pre}}\right)}^{2}}\right]$$

(14) $$FAC2=\frac{X_{pre}}{X_{mea}}$$

(15) $$NMSE=\frac{1}{N}\overset{N}{\underset{i=1}{\sum }}\left(\frac{{\left(X_{mea}-X_{pre}\right)}^{2}}{{\bar{X}}_{mea}.{\bar{X}}_{pre}}\right)$$

where $X_{mea}$ is the measured value of variables; $X_{pre}$ is the predicted value of variables; and ${\bar{X}}_{mea}$ and ${\bar{X}}_{pre}$ are measured and predicted average value of variables, respectively. For the model to be considered adequate, it should meet more than half of the following criteria: |*FB*| < 0.3, 0.7 < *MG* < 1.3, *VG* < 4.0, 0.5 < *FAC2* < 2.0, and *NMSE* < 0.25 [42].

## 3. Results and Discussion

### 3.1. Field Measurement Results

The measured airspeed, temperature, and relative humidity values during summer and winter are summarized in Table 2. In summer, the air temperature, relative humidity, and airspeed values ranged from 24.95 to 26.55 °C, 58.41 to 65.92%, and 0.50 to 2.10 m s^−1^, with the averages of 25.73 °C, 62.90%, and 1.27 m s^−1^, respectively. In winter, the air temperatures ranged from 20.11 to 22.12 °C with an average of 20.90 °C; the air relative humidity ranged from 58.85 to 66.32% with an average of 63.28%, and the airspeeds ranged between 0.18 and 0.57 m s^−1^ with an average of 0.31 m s^−1^. Lindley and Whitaker [14] and Reece and Lott [44] recommended that the optimum indoor temperature of a broiler house should be 32.00–33.00 °C for weeks 1–2 and 21.00–24.00 °C for weeks 3–7. Considering the previous studies, it can be concluded that the temperature of the broiler house on the 40th day was higher than the optimum temperature values in the summer, but remained within the ideal temperature range in the winter. According to Winn and Godfrey [45], the ideal relative humidity for broilers should be between 50 and 70% during rearing periods. Accordingly, it can be stated that the relative humidity values measured in both seasons were within the optimum range. Yahav et al. [46] noted that the optimal airspeed should be 1.5 to 2.0 m s^−1^ for individually housed birds. In the current study, measured airspeed values of broiler house in the winter were under the ideal airspeed values.

### 3.2. Numerical Simulation Results

The mean values of temperature, relative humidity, and airspeed at different heights during the summer and winter are listed in Table 3. In summer, the measured and simulated temperature, relative humidity, and airspeed values were 25.73 ± 0.36 °C and 25.79 ± 1.51 °C, 62.90 ± 2.09% and 59.44 ± 1.32%, and 1.27 ± 0.47 m s*^−^*^1^ and 1.19 ± 0.40 m s*^−^*^1^, respectively. For winter, these values were 20.90 ± 0.50 °C and 20.78 ± 1.24 °C, 63.28 ± 2.35% and 60.03 ± 2.60%, and 0.31 ± 0.09 m s*^−^*^1^ and 0.28 ± 0.10 m s*^−^*^1^, respectively.

**Figure 3:**
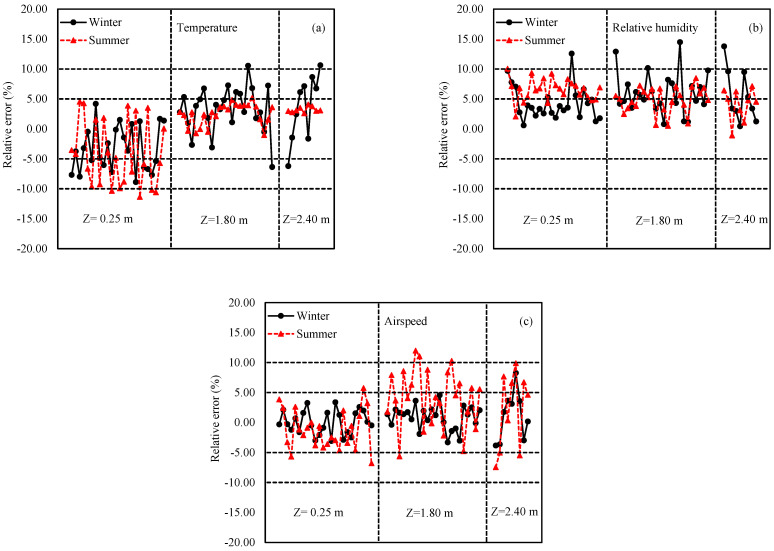
Relative errors of (**a**) temperature, (**b**) relative humidity, and (**c**) airspeed in summer and winter.

The simulated air temperature values at each measurement position were in good agreement with the experimental values for both the summer and winter seasons (Figure 3a). Considering the relative error (%) as a criterion, 25 out of 57 values were higher than or equal to −5% or 5% in winter. Relatively larger discrepancies were found near the fan area, where lower air temperatures were expected. In the summer, 13 out of 57 values were higher than or equal to −5% or 5%, which were also similar to previous reports [27,47].

The relative errors (%) for summer and winter and the comparison of the measured versus predicted air relative humidity values are presented in Figure 3b. In summer, the differences in 35 out of 57 points were higher than or equal to −5% or 5%; in winter, 22 out of 57 values were higher than or equal to −5% or 5%.

It should be noted that the simulations always underestimate the relative humidity at flock height, leading to a positive absolute error. Similar findings have been reported by Du et al. [28], who stated that when the CFD model considered moisture production from birds, manure (not included in this model) would also contribute to the increase in relative humidity. Therefore, the numerical results proved that most of the measured locations showed slightly higher relative humidity than the simulated values.

The results revealed good agreement with the measurements regarding the airspeeds predicted by the CFD model, as shown in Figure 3c. To avoid large relative errors, which can occur when airspeeds are too small, previous studies [23,28,48] usually reported the differences between the measured and the simulated airspeeds as percentages of the mean airspeed at the inlets. This method was also used in this study. In winter, the relative errors remained reasonably stable, probably due to the lower airspeeds in the house caused by the minimum ventilation strategy during cold weather. By contrast, when tunnel ventilation was used in the summer in the simulation, a high discrepancy was observed between the measured and the predicted values at roof levels close to the fan zone, probably related to the increased turbulence. In summer, 21 out of 57 points were higher than or equal to −5% or 5%. Considering the complexity of the air flow, these results can be regarded as satisfactory.

The results showed that the CFD model successfully predicted indoor airspeeds, temperatures, and relative humidity by meeting all criteria (Table 4). The model effectively and efficiently predicted the distribution of indoor environmental parameters and dynamic changes. Although there were some deviations between the simulated and the experimental values, the simulations agreed well with the experimental results.

### 3.3. Evaluation of Indoor Airflow Pattern

**Figure 4:**
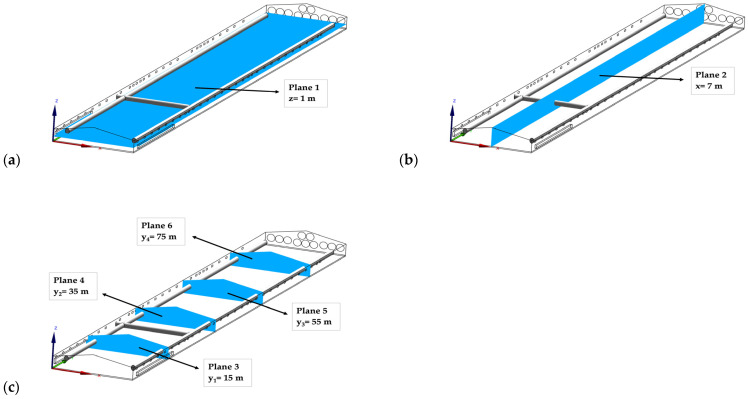
Locations of planes: (**a**) z = 1 m, (**b**) x = 7 m, and (**c**) y_1_ = 15 m, y_2_ = 35 m, y_3_ = 55 m, y_4_ = 75 m, and x = 7 m for illustration of spatial variations of the microclimate.

In the building, six planes were defined to illustrate the spatial variation of the microclimate between winter and summer (Figure 4). Specifically, plane 1 was defined as being at the height of one meter above the ground (z = 1 m), while plane 2 was designed as a longitudinal section of the building (x = 7 m). Planes 3–6 were specified as cross-sections of the building (y_1_ = 15 m, y_2_ = 35 m, y_3_ = 55 m, and y_4_ = 75 m).

#### 3.3.1. Summer Airflow

**Figure 5:**
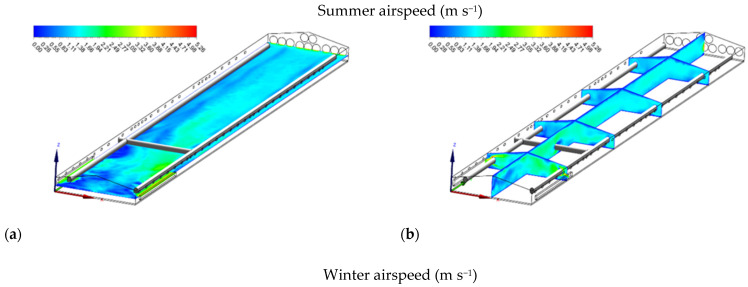

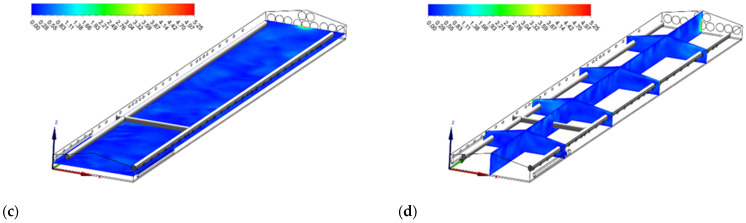
Airspeed (m s^−1^) contours of slice: z = 1 m, and y_1_ = 15 m, y_2_ = 35 m, y_3_ = 55 m, y_4_ = 75 m, and x = 7 m for summer (**a**,**b**) and winter (**c**,**d**).

When tunnel ventilation was simulated for summer to keep the birds cool in hot weather, the airflows were horizontal along the length of the house from the unwetted cooling pads to the exhaust fans in the end walls (Figure 5a). In the summer, the pads could not be wet during the measurement because the sprinkler system was damaged and used as a tunnel inlet opening only to facilitate substantial airflow inside the house. The average airspeeds at the heights of 0.25, 1.80, and 2.40 m above the floor were 0.74 ± 0.15, 1.40 ± 0.27, and 1.58 ± 0.28 m s^−1^, respectively. The largest discrepancy in airspeed was observed at the closing points of the inlets, which was attributable to the high momentum of inlet air from the sidewall tunnel inlet openings. Meanwhile, less circulated zones and draught were also observed near the sidewall inlets. As shown in Figure 5b, the jet moved along the ceiling then backed toward the sidewall at floor level. Therefore, the vortex caused by the rotating air could decrease the uniformity of air distribution.

#### 3.3.2. Winter Airflow

In contrast to summer, simulation in winter conditions showed that it was crucial to prevent cold air from accumulating at flock height when the temperatures were low. During the winter, inlets should be narrowed so that air entered at high pressure and was directed toward the center of the house above the birds for the appropriate mixing of cold (outside) and warm (inside) air (Figure 5c). In the winter simulation, the average airspeeds at the heights of 0.25, 1.80, and 2.40 m above the floor were 0.25 ± 0.07, 0.29 ± 0.10, and 0.37 ± 0.12 m s^−1^, respectively. The maximum airspeed of 0.53 m s^−1^ was found near the fan area, whereas the minimum airspeed of 0.14 m s^−1^ was detected near the front of the house. Compared with summer, the airspeed distributions in winter had less magnitude and variation, attributable to lower ventilation rates and smaller inlet openings. Because the inlets were open at a narrower angle in winter, high airspeed near the inlets created the vortex (Figure 5d). For the same reason, high airspeed near the ceiling was also observed.

Furthermore, there were stagnant zones at flock height close to the house’s front sidewall. In the stagnant zone, heat, moisture, and pollutant gases quickly accumulated, resulting in a poor living environment for the broilers close to the sidewall. Therefore, to create a more comfortable living environment for the broilers in the stagnant zones, additional partial ventilation systems (i.e., mixing fans, ceiling fans), which would increase the local air exchange rate, are highly recommended [16,49].

### 3.4. Evaluation of Indoor Thermal Environment

#### 3.4.1. Temperature

**Figure 6:**
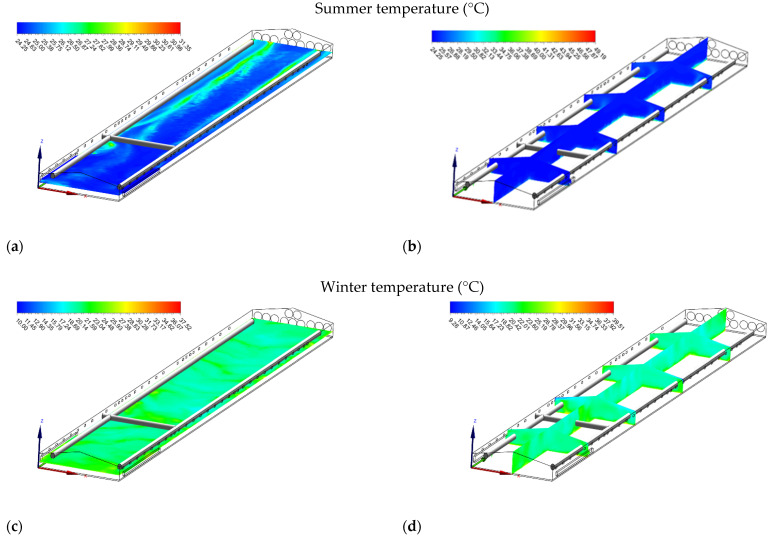
Temperature (°C) contours of slice: z = 1 m, and y_1_ = 15 m, y_2_ = 35 m, y_3_ = 55 m, y_4_ = 75 m, and x = 7 m for summer (**a**,**b**) and winter (**c**,**d**).

In summer, there was a significant increase in temperature values from air inlets to the exhaust fans; as the air flowed along the length of the house, it carried the heat generated by the broilers (Figure 6a). The average air temperatures at the heights of 0.25, 1.80, and 2.40 m above the floor were 27 ± 1.58, 25 ± 0.62, and 24.66 ± 0.21 °C, respectively. The highest air temperature (27.75 °C) was found near the exhaust fan, whereas the lowest (24.45 °C) was observed near the tunnel inlet openings (Figure 6b). At the end of the summer grow-out, the air temperature was not within the ideal limit because the recommended temperatures for broilers are between 18 and 21 °C [50]. Increasing ventilation is one of the practical ways to keep the houses and birds from overheating in hot weather. However, keeping at the maximum ventilation alone may still be insufficient in some hot weather conditions, and evaporative cooling is needed. In hot weather, operating a well-maintained ventilation system with evaporative cooling can be considered among the possible solutions to reduce heat stress [13,16].

Similarly, air temperature during winter increased from the front through to the end of the house due to the transportation of heat throughout the house (Figure 6c). The average air temperatures at the heights of 0.25, 1.80, and 2.40 m above the floor were 21.82 ± 0.75, 20.01 ± 0.78, and 20.06 ± 1.32 °C, respectively. The highest air temperature (22.35 °C) was found near the exhaust fan, whereas the lowest air temperature (19.15 °C) was observed near the front wall (Figure 6d).

#### 3.4.2. Relative Humidity

**Figure 7:**
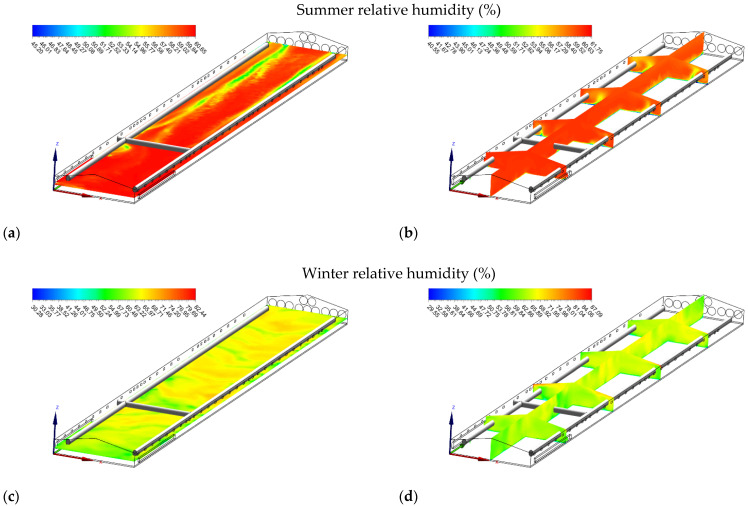
Relative humidity (%) contours of slice: z = 1 m, and y_1_ = 15 m, y_2_ = 35 m, y_3_ = 55 m, y_4_ = 75 m, and x = 7 for summer (**a**,**b**) and winter (**c**,**d**).

The air relative humidity distributions in the house in summer are shown in Figure 7a,b. In summer, the lowest relative humidity values (<55%) were near the house’s end sidewall, where the highest air temperatures were observed. Higher relative humidity values (>60%) were found near the front of the house where the lowest air temperatures occurred. In the winter season, the highest relative humidity values (>60%) were found near the front of the house, where generally the lowest temperature values were detected (Figure 7c). Furthermore, the highest relative humidity values (>87%) increased along with the ceiling near the inlets due to higher outside relative humidity (Figure 7d).

The simulation of the original design indicated that sufficient cooling could not be achieved in the summer period. Additionally, there were stagnant zones in the winter period. Having recognized the environmental problems in the house in summer and winter, CFD models were designed to study these problems and design improvements, which are presented in Section 3.5.

### 3.5. Design Improvement

#### 3.5.1. Summer Cooling with Evaporative Pad

**Figure 8:**
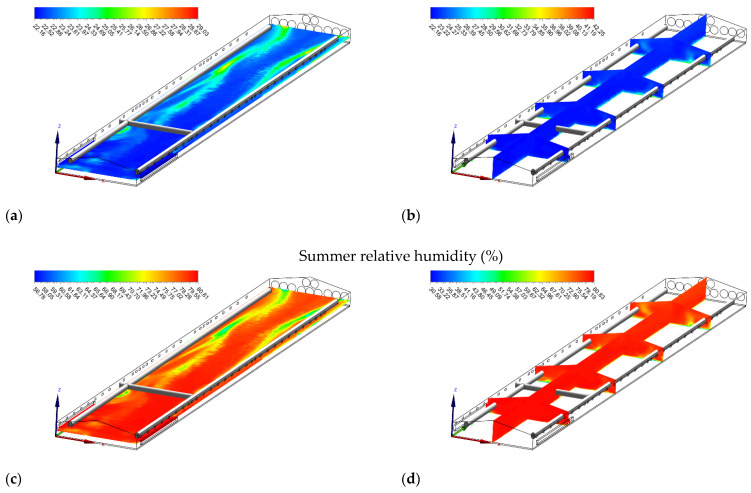
Temperature (°C) (**a**,**b**) and relative humidity (%) (**c**,**d**) contours of slice: z = 1 m, and y_1_ = 15 m, y_2_ = 35 m, y_3_ = 55 m, y_4_ = 75 m, and x = 7 m in summer when operating evaporative cooling pads.

In summer, air temperatures in the broiler house were higher than the acceptable limits. The simulation results demonstrated that when an evaporative cooling system was used in summer, a cooling of approximately 3 °C could be achieved in the house. Additionally, while the air temperatures were low (22.16–22.88 °C) near the pads, they gradually increased by almost 2.50 °C from the pads to the exhaust fans (Figure 8a,b). Numerous studies have revealed that evaporative cooling pad systems may provide a solution for controlling the high temperatures that negatively affect the poultry buildings [51,52,53]. However, although the evaporative cooling pad system significantly decreases indoor temperatures, it also causes a significant increase in relative humidity inside the house (Figure 8c,d), which can be problematic, especially in humid climates [16].

#### 3.5.2. Removal of Stagnant Zones with Circulation Fans

**Figure 9:**
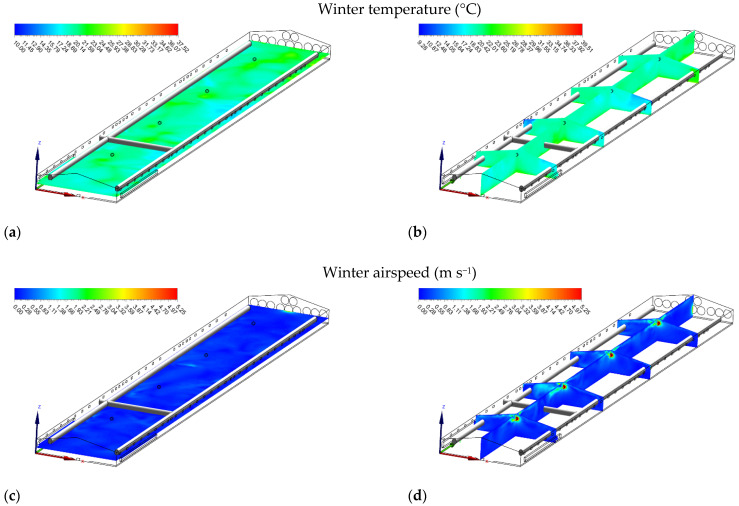
Temperature (°C) (**a**,**b**) and airspeed (m s^−1^) (**c**,**d**) contours of slice: z = 1 m, and y_1_ = 15 m, y_2_ = 35 m, y_3_ = 55 m, y_4_ = 75 m, and x = 7 m in winter when installing circulation fans.

In winter, low airspeed resulted in the formation of “stagnant zones.” When adding four 500-mm circulation fans (Hydor Ltd., Salisbury, UK) of 20-m spacing inside the poultry housing and using the same initial and boundary conditions, the numerical study results showed that the accumulation of hot and humid air in the ceiling of the house was eliminated (Figure 9a,b). More homogenous air distribution throughout the house was achieved, and the stagnant areas were decreased in the house (Figure 9c,d). These results agreed with Bottcher et al. [54], who reported a decrease in temperature in the horizontal and vertical axes of the poultry building, resulting in significant savings in heating costs.

## 4. Conclusions

This study was conducted to simulate the summer and winter microclimates in a mechanically ventilated broiler house. The following conclusions were drawn from the results.

Combining field measurements and numerical modeling proved that the application of CFD facilitated the identification of environmental issues in the broiler house that could affect broilers’ development and simulate effective solutions.

In summer, if an evaporative cooling pad system was used, a decrease in temperature of approximately 3 °C could be achieved when the mean air temperature rose above 25 °C in the house. In winter, adding four 500-mm circulation fans of 20-m spacing inside the house could eliminate the accumulation of hot and humid air in the stagnant zones in the house, resulting in the homogenous spatial distribution of air in the house.

This study demonstrated that CFD application in future research might allow more realistic solutions by modeling the conditions under which broilers are reared.

## Figures and Tables

**Table 1:** Boundary conditions in this study.

Element	Summer	Winter
Number of birds (n)	17,684	17,364
Live weight (g)	2640	2340
*SHP* (W bird^−1^)	23.740	18.270
*LHP* (W bird^−1^)	16.238	11.380
*MP* (kg s^−1^)	6.628 × 10^−6^	4.645 × 10^−6^
T_inlet_ (°C)	29.12	8.80
RH_inlet_ (%)	48.33	81.30
V_inlet_ (m s^−1^)	5.80	4.63
Inlet opening angle (°)	45.00	22.50
Number of operating fans (n)	9	2

Abbreviations: *SHP*, sensible heat production; *LHP*, latent heat production; *MP*, moisture production; T_inlet_, inlet temperature; RH_inlet_, inlet relative humidity; V_inlet,_ inlet airspeed.

**Table 2:** Descriptive statistics for the temperature, relative humidity, and airspeed.

Seasons	Parameters	Min	Max	Mean	SD	Sk	Kr
Summer	Temperature (°C)	24.95	26.55	25.73	0.36	0.19	−0.74
Winter	Temperature (°C)	20.11	22.12	20.90	0.50	0.81	−0.30
Summer	Relative humidity (%)	58.41	65.92	62.90	2.09	−0.50	−0.83
Winter	Relative humidity (%)	58.85	66.32	63.28	2.35	−0.25	−1.60
Summer	Airspeed (m s^−1^)	0.50	2.10	1.27	0.47	−0.12	−1.54
Winter	Airspeed (m s^−1^)	0.18	0.57	0.31	0.09	1.00	0.70

Abbreviations: Min, minimum; Max, maximum; SD, standard deviation; Sk, skewness; Kr, kurtosis.

**Table 3:** Measured and simulated temperature, relative humidity, and airspeed values (mean ± standard deviation).

Parameters	Height	Summer		Winter	
		Measured	Simulated	Measured	Simulated
Temperature (°C)	0.25 m	25.92 ± 0.31	27.00 ± 1.58	21.14 ± 0.49	21.82 ± 0.75
	1.80 m	25.63 ± 0.34	25.00 ± 0.62	20.69 ± 0.43	20.01 ± 0.78
	2.40 m	25.48 ± 0.26	24.66 ± 0.21	20.81 ± 0.47	20.06 ± 1.31
	All	25.73 ± 0.36	25.79 ± 1.51	20.90 ± 0.50	20.78 ± 1.24
Relative humidity (%)	0.25 m	63.53 ± 2.07	59.34 ± 1.42	60.79 ± 0.75	58.18 ± 1.72
	1.80 m	62.36 ± 2.06	59.31 ± 1.41	65.19 ± 0.72	61.27 ± 2.20
	2.40 m	62.68 ± 2.03	60.05 ± 0.41	65.27 ± 0.73	61.66 ± 2.66
	All	62.90 ± 2.09	59.44 ± 1.32	63.28 ± 2.35	60.03 ± 2.60
Airspeed (m s^−1^)	0.25 m	0.76 ± 0.13	0.83 ± 0.22	0.25 ± 0.05	0.25 ± 0.08
	1.80 m	1.62 ± 0.18	1.40 ± 0.27	0.33 ± 0.08	0.29 ± 0.10
	2.40 m	1.58 ± 0.36	1.58 ± 0.21	0.42 ± 0.10	0.37 ± 0.12
	All	1.27 ± 0.47	1.19 ± 0.40	0.31 ± 0.09	0.28 ± 0.10

**Table 4:** Statistical parameters for model performance evaluation.

Seasons	Parameters	*FB * (<0.3)	*FAC2 * (0.5–2.0)	*MG * (0.7–1.3)	*VG * (<4.0)	*NMSE* (<0.25)
Summer	Temperature	0.002	1.002	0.998	0.998	0.003
	Relative humidity	0.057	0.945	1.058	1.119	0.004
	Airspeed	0.066	0.974	1.056	1.115	0.053
Winter	Temperature	0.006	0.994	1.007	1.014	0.003
	Relative humidity	0.054	0.948	1.056	1.114	0.004
	Airspeed	0.082	0.964	1.105	1.220	0.146

Abbreviations: *FB*, fractional bias; *FAC2*, fraction within a factor of two; *MG*, geometric mean bias; *VG,* geometric mean-variance; *NMSE*, normalized mean square error.

## Data Availability

Not applicable.
